# Awareness and Attitudes Toward Childhood Mental Disorders Among Primary School Teachers in Taif, Saudi Arabia

**DOI:** 10.7759/cureus.49377

**Published:** 2023-11-25

**Authors:** Anas Ibn Auf, Ziyad S Alharthi, Abdulaziz I Almalki, Abdullah E Alharbi, Abdullah Alaqla, Bader M Alharthi, Ahmed S Alsaedi, Alaa H Alhabib

**Affiliations:** 1 Psychiatry, Erada and Mental Health Complex, Taif, SAU; 2 Psychiatry, Eastern Sudan College for Medical Sciences and Technology, Port Sudan, SDN; 3 Medicine, King Abdulaziz University Faculty of Medicine, Rabigh, SAU

**Keywords:** mental health, psychiatry, saudi arabia, taif, teachers, primary school, childhood mental disorders, awareness, attitudes

## Abstract

Background and aim

Early detection and intervention can improve the treatment outcome of childhood mental disorders, and primary school teachers may play an important role in referring suspected cases to mental health facilities if they have good awareness and attitudes toward these disorders. The aim of this study is to assess the awareness and attitudes of primary school teachers toward childhood mental disorders in Taif, Saudi Arabia.

Methods

This is a cross-sectional study conducted among classroom teachers in primary schools in Taif, Kingdom of Saudi Arabia. It was conducted during the period from 2022 to 2023 in both public and private schools. An anonymous, self-administered, online questionnaire was used to assess participants’ awareness and attitudes toward pupils with mental health issues. The collected data were analyzed using the chi-square test to examine the associations between various categories and the ANOVA test to compare means.

Results

The study included 417 teachers, 63.5% of whom were males, the mean of their ages was 39.59 years (SD±8.66), and the mean of their work experience was 12.8 years (SD±8.02) in different teaching specialties. Among participants, 60.2% claimed that no pupils had mental health problems in their classes, 80.1% had not referred any pupils to mental health facilities, and 88.5% did not receive any training related to childhood mental health problems. A humble percent (12.2%) of the participants claimed a good awareness of the signs and symptoms of childhood disorders. Only 54% of teachers advise visiting a psychiatric clinic in case of psychiatric problems, and a similar percentage of teachers believe that psychiatric drugs cause addiction. The male gender, being specialized in humanitarian subjects, having relatives or friends with childhood mental disorders, and receiving training related to childhood mental health were significantly associated with teachers’ better awareness.

Conclusion

Primary school teachers generally lack awareness of childhood mental health and have underestimation and poor recognition of cases of mental disorders. There are many teachers who also have unfavorable attitudes toward psychiatric disorders, patients, and treatments, which requires much effort to improve their awareness and attitudes toward childhood mental disorders.

## Introduction

Mental problems among children are common and may have significant long-term health biopsychosocial consequences [1]. More than one-third of mental problems appear in children before the age of 14 years [2]. Mental illnesses are often looked upon as weakness and are neglected unless the symptoms are severe [3]. Visiting a mental health expert is left to be the last option [4], and this leads to late diagnosis, which results in poor prognosis or many complications such as self-harm or suicide, which could have been avoided if treated early [5]. The majority of these children are school-aged and most of them are pupils, they spend a lot of their daily time at school especially primary school pupils which to them is the second place after home where they take an impression of outside society, and these children are under the observation and guidance of their teachers who are trusted and respected by the children.

Teachers have a unique position in monitoring and detecting pupils with childhood mental disorders who have clinical or subclinical symptoms [6]. In addition, teachers interact daily with a large group of pupils and can detect early signs of mental health disorders if they are aware of the symptoms and signs of these disorders. They can also speed the detection of mental health disorders by assisting the referral to a mental health professional if they have favorable attitudes toward these disorders and a tendency to help those pupils. This can assist in early detection and proper treatment [7,8].

In Saudi Arabia, previous studies showed a suboptimal level of knowledge of attention deficit hyperactivity disorder (ADHD) among primary school teachers in Riyadh [9], Medina [10], and Abha [11]. In addition, in Al-Badaya city, school teachers had a low level of knowledge about autism spectrum disorder [12]. A study claims that 38% of teachers in Saudi Arabia had inadequate knowledge about ADHD [10]. Other research concluded that there isn’t enough knowledge among teachers about mental health issues, although it is supposed that teachers play a significant role in the detection, prevention, and screening of these childhood problems aiming to guard against negative future consequences [13].

The importance of this research is that it helps in the promotion of the pupil’s mental health by assessing the teachers’ awareness and attitudes toward childhood mental health. To the knowledge of the researchers, there aren’t many research studies dealing with the same issue in our region. In this study, we aim to assess the attitudes of primary school teachers toward childhood mental disorders in view of the problem and the importance of early detection and referral for effective treatment.

## Materials and methods

Study design and location

A cross-sectional study was conducted among classroom teachers in primary schools in Taif, Kingdom of Saudi Arabia. Taif is one of the largest governorates in the Makkah region which is located in the western part of the Kingdom of Saudi Arabia. It represents a large area of the Makkah Region estimated at 42,750 square kilometers, with a population of 913,374 [14]. The research was conducted during the period from 2022 to 2023 and was approved by the Directorate of Health Affairs - Taif, Research and Studies Department (approval number: 662).

Participants

Classroom teachers at primary schools in Taif were the target population of the study. Primary schools in Saudi Arabia enroll children between the ages of 6 and 12.

Sample size and sampling technique

To ensure representativeness, schools were systematically randomized from a list of all schools in the governorate, including both public and private schools, and the questionnaire link was sent to the selected schools. The study targeted teachers of both genders who have at least one year of experience in teaching. The sample size was calculated according to the following equation: (S=Z2 *p*(1-p)/M2), where S is the sample size for an infinite population, Z is the Z score of 1.96, P is the population proportion (assumed to be 50%, 0.5), and M is the margin of error of 5% (0.05). The sample size (S) based on this formula is 384. About 10% were added to guard against non-response.

Data collection tool

An anonymous, self-administered, online questionnaire was used to assess participants’ awareness and attitudes toward pupils with mental health issues. The questionnaire content was obtained from a previous study done by Kamel et al. for a similar aim in Hail Governorate, Saudi Arabia, in 2020 [13]. It consisted of four parts: The first included socio-demographic and characteristic factors of teachers. The second part included seven questions with a 5-point Likert scale ranging from 1 (not aware at all) to 5 (completely aware), which was designed to evaluate teachers’ levels of awareness about pupil mental issues. The third part consisted of 11 questions with a 5-point Likert scale ranging from 1 (completely disagree) to 5 (completely agree) in order to evaluate teachers’ attitudes toward mental health issues. The last part included suggested ways to raise awareness of childhood mental issues, and teachers were asked to rate the importance of each of them on a scale ranging from 1 (not important at all) to 5 (very important).

Statistical analysis

The SPSS Statistics version 24 software (IBM Corp. Released 2016. IBM SPSS Statistics for Windows, Version 24.0. Armonk, NY: IBM Corp.) was used to analyze the collected data. Descriptive data were presented in frequencies and percentages, and the ANOVA test was used to compare means. The chi-square test was used to analyze the associations among various categories, and a p-value of <0.05 was considered statistically significant.

## Results

The study included 417 teachers who completely answered the questionnaires. About two-thirds of them (63.5%) were male participants and the rest (36.5%) were female. The mean of their ages was 39.59 years (SD±8.66), and they were distributed according to their age groups in Table 1. Of the participants, just below half (47.2%) had 1-10 years of work experience, 33.1% had 11-20 years, and the rest (19.7%) had 20 years or more of work experience with a mean of 12.8 years (SD±8.02). Moreover, a majority (N=327, 78.4%) of the participants were married, while unmarried (singles, divorced, or widowed) constituted 21.5%. Likewise, significant proportions (388, 93%) of the participants had a bachelor's degree. Regarding specialties, 47% of the participants (196) were teaching humanitarian subjects (language, art, religion, etc.), whereas 30.5% (127) were teaching science and mathematics subjects (Table 1).

**Table 1:** Distribution of the study sample according to socio-demographic and other characteristic factors

Characteristics of teachers		Frequency	Percent
Sex	Male	265	36.5%
	Female	152	36.5%
Age	25-34 years	124	29.7%
	35-44 years	171	41%
	45-54 years	98	23.5%
	55-64 years	24	5.8%
Teaching experience	1-10 Years	197	47.2%
	11-20 Years	138	33.1%
	>20 Years	82	19.7%
Marital status	Single	64	15.3%
	Married	327	78.4%
	Divorced	18	4.3%
	Widowed	8	1.9%
Qualification	Diploma	9	2.2%
	Bachelor	388	93 %
	Master	20	4.8%
Teaching specialty	Humanitarian subjects	196	47%
	Scientific subjects	127	30.5%
	Other	94	22.5%
School type	Public	368	88.2%
	Private	49	11.8%
Average pupils per class	<20	65	15.8%
	20-30	195	46.8%
	>30	165	37.4%
Average number of pupils with psychiatric problems per class (estimated by teacher)	None	251	60.2%
	1-2	141	33.8%
	>2	25	6%
Average pupils referred to mental health facilities	None	334	80.1%
	One	36	8.6%
	>1	47	11.3%
Having relatives with childhood mental disorders	Yes	189	45.3%
	No	228	54.7%
The degree of relationship	1: children or siblings	25	6.5%
	2: nephews or nieces	73	17.5%
	3: cousins or uncle girls	70	16.8%
	4: others	21	5%
Having friends who had childhood mental disorders	Yes	195	46.8%
	no	222	53.2%
Receiving any training related to childhood mental disorders	Yes	48	11.5%
	no	369	88.5%
Total		417	100%

The estimation of pupils’ numbers per class is shown in Table 1. More than half of the participants (251, 60.2%) claimed that no pupils had mental health problems in their classes, and one-third of the participants (141, 33.8%) reported that there were one to two pupils with mental health problems in each class. The majority of the participants (80.1%) had not referred any pupils to mental health facilities (Table 1). Among the study sample, 45.3% (189) had relatives with childhood mental disorders, and 46.4% (195) had friends with childhood mental disorders. A clear majority of the participants (88.5%) did not receive any training related to childhood mental health problems (Table 1).

Regarding teachers’ awareness of pupils’ mental issues, only 10.3% of the participants (N=43) answered all seven questions with "aware\completely aware." Table 2 presents the numbers and percentages of teachers according to the detailed aspects of awareness. Regarding knowledge about the risk factors of mental health, around 40% (M=2.75, SD=1.046) answered "somewhat aware" and only 22.5% had enough information. Similar results were found regarding the causes of the mental health issues: around 38% (M=2.72, SD=1.040) answered "somewhat aware" and about a fifth of teachers had enough information. A good number of the participants claimed to have enough information about the signs and symptoms of childhood mental disorders (42.4%, M=3.18, SD=1.165). More than half of the participants (53%, M=2.41, SD=1.123) reported inadequate or poor knowledge concerning psycho-behavioral interventions, and 46.5% (M=2.59, SD=1.104) of teachers were not aware of the system dealing with psychiatric patients. Teachers who were aware of school psychological health services available to pupils were 22.8% (M=2.62, SD=1.119), and those who were aware of community mental health services were 24.3% (M=2.72, SD=1.134). See Table 2.

**Table 2:** Teachers' awareness of childhood mental disorders

Statement	Responses: n (%)					Score mean (SD)
	Not aware at all	Not aware	Somewhat aware	Aware	Completely aware	
Risk factors of childhood mental disorders	59 (14.1%)	97 (23.3%)	167 (40%)	76 (18.2%)	18 (4.3%)	2.75 (1.046)
Causes of childhood mental disorders	60 (14.4%)	105 (25.2%)	159 (38.1%)	78 (18.7%)	15 (3.6%)	2.72 (1.040)
Psycho-behavioral therapy	111 (26.6%)	110 (26.4%)	129 (30.9%)	50 (12%)	17 (4.1%)	2.41 (1.134)
Community mental health services	76 (18.2%)	91 (21.8%)	149 (35.7%)	77 (18.5%)	24 (5.8%)	2.72 (1.134)
System dealing with psychiatric patients	81 (19.4%)	113 (27.1)	139 (33.3%)	65 (15.6%)	19 (4.6%)	2.59 (1.104)
Signs and symptoms of childhood mental disorders	46 (11%)	63 (15.1%)	131 (31.4%)	126 (30.2%)	51 (12.2%)	3.18 (1.165)
School psychological health services available to pupils	76 (18.2%)	123 (29.5%)	123 (29.5%)	75 (18%)	20 (4.8%)	2.26 (1.119)

As shown in Table 3, the majority of teachers (86.8%, M=1.67, SD=0.734) believed in psychiatric disorders, and 86.4% (M=1.69, SD=0.767) claimed accepting psychiatric patients and helping them. More than half of teachers 54% (M=1.68, SD=0.913) advised visiting a psychiatric clinic in case of psychiatric problems, and at the same time, 54% of teachers (M=2.52, SD=1.096) believed that psychiatric drugs cause addiction. A minority of them, 17.9% (M=3.60, SD=1.169), believed that psychiatric diseases were only caused by magic and the evil eye. Two-thirds of teachers believed that narcotics, stimulant drugs, and alcohol have a role in psychiatric disorders, and less than half believed that heredity plays a role in psychiatric disorders. Only 45.8% of teachers thought that psychiatric patients were the same as any other organic disease patients and only 40% disagreed with the belief that mental health illnesses are incurable. More than half (54.2%) of teachers didn't believe that a psychiatric disorder is a permanent stigma, but 35% considered psychiatric patients as a danger and must be dealt with cautiously.

**Table 3:** Teachers' attitudes toward childhood mental disorders

Statement	Responses: n (%)					Score mean (SD)
	Completely disagree	Disagree	Not sure	Agree	Strongly agree	
I believe in psychiatric disorders.	1 (0.2)	3 (0.7%)	51 (12.2%)	165 (39.6)	197 (47.2%)	1.67 (0.734)
I accept mental health patients and I help them.	1 (0.2%)	8 (1.9%)	48 (11.5%)	165 (39.6)	195 (46.8%)	1.69 (0767)
When having a mental health problem, I recommend visiting a psychiatric physician.	7 (1.7%)	10 (2.4%)	56 (13.4%)	113 (27.1%)	231 (55.4%)	1.68 (0.913)
I see that psychiatric medications cause addiction.	28 (6.7%)	41 (9.8%)	123 (29.5)	152 (36.5%)	73 (17.5%)	2.52 (1.096)
I believe that mental illness is merely a mix of magic and eye.	127 (30.5%)	87 (20.9%)	128 (30.7%)	59 (14.1%)	16 (3.8%)	3.60 (1.169)
I think narcotics, stimulant drugs, and alcohol have a role in psychiatric illness.	6 (1.4%)	24 (5.8%)	105 (25.2%)	176 (42.2%)	106 (25.4%)	2.16 (0.918)
I believe that heredity has a role in mental health illness.	15 (3.6%)	33 (7.9%)	187 (44.8%)	115 (27.6%)	67 (16.1%)	2.55 (0.972)
I see mental health patients as other patients with organic diseases.	21 (5%)	33 (7.9%)	172 (41.2%)	132 (31.7%)	59 (14.1%)	2.58 (0.995)
I believe that mental health illnesses are incurable.	72 (17.3%)	97 (23.3%)	146 (35%)	75 (18%)	27 (6.5%)	3.27 (1.137)
I believe that mental health illness is a permanent stigma.	113 (27.1%)	113 (27.1%)	98 (23.5%)	67 (16.1%)	26 (6.2%)	3.53 (1.1221)
I believe that a psychiatric patient is a dangerous person and one should be taken with care.	56 (13.4%)	59 (14.1%)	154 (36.9%)	120 (28.8)	28 (6.7%)	2.99 (1.113)

ANOVA, as shown in Table 4, was applied to determine factors associated with teachers' better awareness. It revealed that male gender (p-value =0.002), being specialized in humanitarian subjects (p-value =0.001), having relatives or friends with childhood mental disorders (p-value <0.001), receiving training related to childhood mental health (p-value <0.001), and reporting higher numbers of mental health problems among pupils (p-value <0.001) and referring them to mental health facilities (p-value <0.001) were significantly associated with teachers' better awareness. Regarding attitudes, receiving training related to childhood mental health (p-value <0.001) and reporting higher numbers of mental health problems among pupils (p-value =0.001) were significantly associated with teachers’ favorable attitudes.

**Table 4:** The association between teachers’ awareness, attitudes, and their characteristics

Variables		Awareness			Attitude		
		Mean	N	p-value	Mean	N	p-value
Age	25-34	19.1 (5.9)	124	0.065	40.2 (5.5)	124	0.260
	35-44	18.8 (5.9)	171		39.6 (5.2)	171	
	45-54	18.4 (6.9)	98		39.0 (6.7)	98	
	55-64	22.0 (4.4)	24		38.1 (4.1)	24	
Teaching experience	1-10 years	18.5 (5.9)	197	0.104	39.6 (5.9)	197	0.978
	11-20 years	18.9 (6.3)	138		39.6 (5.4)	138	
	>20 years	20.2 (6.1)	82		39.5 (6.6)	82	
Sex	Male	19.7 (6.2)	256	0.002	39.8 (5.8)	265	0.354
	Female	17.8 (5.7)	152		39.2 (5.2)	152	
Marital status	Single	18.7 (6.1)	64	0.858	38.7 (5.4)	64	0.432
	Married	18.9 (6.1)	327		39.8 (5.6)	327	
	Divorced	19.8 (7.4)	18		38.7 (7.0)	18	
	widowed	20.3 (5.5)	8		39.0 (4.5)	8	
Qualification	Diploma	17.1 (5.8)	9	0.169	39.0 (6.5)	9	0.514
	Bachelor	18.9 (6.2)	388		39.5 (5.6)	388	
	Master	21.2 (4.5)	20		40.9 (5.2)	20	
Teaching specialty	Humanitarian subjects	20.0 (5.7)	196	0.001	40.1 (5.7)	196	0.146
	Scientific subjects	18.7 (6.1)	127		39.2(5.7)	127	
	Other	17.3 (6.6)	94		38.9(5.7)	94	
School type	Public	19.2 (6.2	368	0.059	39.7(5.7)	368	0.146
	Private	17.1 (5.4)	49		38.5(5.4)	49	
Having relatives with childhood mental disorders	Yes	20.3 (5.4)	189	<0.001	39.8(5.3)	189	0.546
	No	17.9 (6.5)	228		39.4(5.9)	228	
Having friends who had childhood mental disorders	Yes	20.2 (5.3)	195	<0.001	39.9(5.4)	195	0.249
	No	17.9 (6.6)	222		39.3(5.9)	222	
Receiving training related to childhood mental disorders	Yes	26.9 (3.9)	48	<0.001	44.1(5.8)	48	<0.001
	No	17.9 (5.5)	369		38.9(5.3)	369	
Average pupils estimated by the teacher to have psychiatric problems (per class)	None	17.9 (6.3)	251	<0.001	38.9(5.6)	251	0.001
	1-2	20.1 (5.4)	141		40.3(5.4)	141	
	3 or more	22.9 (5.1)	25		42.5(6.4)	25	
Referring pupils to mental health facilities	No	18.3 (5.9)	334	<0.001	39.4(5.5)	334	0.260
	yes	21.6 (6.2)	83		40.2(5.9)	83	

As shown in Table 5, which presents teachers’ ratings of the suggested ways of raising awareness of mental health issues, almost two-thirds of teachers found the training programs targeting teachers to be important. A majority of the teachers (80.4%) believe in the importance of providing a psychologist service in schools. Two-thirds found the importance of conducting workshops about mental health in school for all staff, and similarly, 68.3% found the importance of equipping schools with prints and audio-visual materials discussing childhood mental health. The percentages of those who considered the importance of "providing a telephone hotline for mental problems" and "increasing awareness by general media" were 81.8% and 88%, respectively.

**Table 5:** Suggested ways to raise awareness of childhood mental issues (as perceived by teachers)

Statement	Responses: n (%)					Score mean (SD)
	Not important at all	Not important	Somewhat important	Important	Extremely important	
Training programs for teachers	12 (2.9%)	3.86 (1.012)	126 (30.2%)	127 (30.5%)	136 (32.6%)	3.86 (1.012)
Availing psychologists in schools to provide mental health counseling services	11 (2.6%)	4.28 (1.036)	49 (11.8%)	92 (22.1%)	243 (58.3%)	4.28 (1.036)
Workshops in mental health for all school staff	4 (1%)	3.93 (0.922)	118 (28.3)	144 (34.5%)	134 (32.1%)	3.93 (0.922)
Availing audiovisual and print materials at schools	11 (2.6%)	3.94 (0.993)	105 (25.2%)	141 (33.8%)	144 (34.5%)	3.94 (0.993)
Providing telephone hotlines to deal with acute mental health problems	5 (1.2%)	4.44 (0.848)	32 (7.7%)	112 (26.9%)	256 (61.4%)	4.44 (0.848)
General media should increase community awareness of mental health	4 (1%)	4.18 (0.841)	62 (14.9%)	171 (41%)	170 (40.8%)	4.18 (0.841)

## Discussion

Teachers may play a fundamental role in the lives of their pupils through early recognition of their mental health problems and early referral to specialized healthcare services. This study focused on teachers’ awareness and attitudes toward childhood mental health problems and showed some significant results.

As shown in the study, one-third of participants (33.8%) estimated the number of pupils with mental health problems in each class to be one to two, and only 6% of teachers reported that the number is more than two pupils. The others claimed that there were no pupils with mental health problems in their classes. This shows that the teachers’ estimation of the number of pupils who had mental health problems is very low. The actual prevalence of childhood mental disorders is far higher than what was estimated by our teachers. A recent systematic review and meta-analysis of the prevalence of mental disorders in children and adolescents was done by Sacco et al. in 2022. It revealed that the pooled prevalence of mental disorders among children was 15.5% (CI 95% 9.4-24.5) [15]. A study from Al Ain in the United Arab Emirates concluded that 22% is the prevalence [16], and some local studies in the Kingdom of Saudi Arabia found that the prevalence is 36.3% [17]. Teachers’ underestimation and poor recognition of mental health problems in our study are in harmony with some previous research [18,19]. It seems that teachers’ underestimation of the prevalence of childhood mental disorders in their classes reflects their poor awareness and knowledge regarding the signs and symptoms of these disorders. This is also supported by the results in Table 4, which showed a statistically significant association between teachers’ awareness and the average number of pupils estimated by the teacher to have psychiatric problems in each class. According to the same table, those who referred pupils to mental health facilities were found to have a better awareness of childhood mental disorders. This is similar to what was concluded by a previous study in the Kingdom of Saudi Arabia in Hail [13]. It proved that lack of awareness of psychiatric problems is among the factors that reduce teachers’ tendency to provide help or refer pupils to receive help [20,21].

Our study showed that teachers do not have adequate awareness and knowledge of different aspects of mental health, especially psychological health services and therapeutic interventions. This highlights the urgent need to design and implement effective interventions aimed at raising teachers’ awareness of mental health.

Teachers in our study seem to have favorable attitudes toward psychiatric disorders and patients. The majority support visiting a psychiatrist when having a mental health problem, do not believe that mental illness is merely a mix of magic and the evil eye, and think narcotics, stimulant drugs, and alcohol have a role in psychiatric illness. On the other hand, teachers’ answers to some of the questions reflected negative attitudes; about 84% of the participants believed or were uncertain that psychiatric medications lead to addiction. This is consistent with previous local research [13], and this is of utmost importance to be corrected since it is likely to decrease teachers’ inclination to refer their pupils to mental health services to avoid assumed drug addiction. Only 45.8% of teachers think that psychiatric patients are the same as any other organic disease patients, and 35% consider psychiatric patients a danger that must be dealt with cautiously. These negative beliefs are common among teachers and the general population [22,23], and they reinforce the stigma of mental illnesses, which decreases the likelihood of helping children with mental health problems or trying to understand their condition.

According to our study, male teachers had better awareness compared to females, and this is similar to what was reported by Kamel et al. [13]. It can be justified by the social and cultural background of the participants from the Taif region where male teachers have more chances to receive educational courses and be enrolled in training programs. Teachers who specialize in humanitarian subjects showed better awareness, which may be expected since they have more knowledge in psychology and social sciences. Having relatives or friends with childhood mental disorders was associated with better awareness, and this is likely to be due to previous exposure to the information and experience of dealing with former or current psychiatric patients. Training related to childhood mental health was significantly associated with teachers’ better awareness and attitudes, confirming the importance of implementing training programs addressing these issues.

Most of the study participants agreed on the importance of the six suggested ways to raise awareness of childhood mental disorders that reflect the need for interventions and teachers' willingness to benefit from them. The suggested interventions according to their perceived importance are providing telephone hotlines to deal with acute mental health problems, availing psychologists in schools for counseling services, using general media, availing audiovisual and print materials at schools, and arranging workshops and training programs for teachers.

Limitations of the study

This study has some limitations, starting with the whole sample being taken from one area, the Taif governorate, which limits the study's generalizability. As most quantitative studies use online questionnaires, answers are closed-ended, and there is no chance for the participants to elaborate more. Although this facilitates conducting the study, it limits the amount of information and views obtained from the participants.

## Conclusions

The study showed that teachers in Taif generally lack awareness of many aspects of childhood mental health. Although some of them claimed to have enough knowledge about the signs and symptoms of childhood mental disorders, they underestimated and poorly recognized the cases among their pupils. A considerable percentage of teachers also have unfavorable attitudes toward psychiatric disorders, patients, and treatments. Half of them do not advise visiting a psychiatric clinic in cases of psychiatric problems. One of the important factors that improve their awareness and attitudes is receiving training courses on mental health issues.

This study described teachers’ awareness and attitudes toward childhood mental disorders at Taif schools and the suggested ways of improvement. It is recommended that much effort be paid to raise the teachers’ knowledge in this area to increase their ability to early recognize and refer suspected cases. This will help our children's mental health and ensure their bright future.
